# The Relationship Between the C-reactive Protein–Triglyceride Glucose Index and the Risk of Coronary Heart Disease Alongside the Severity of Coronary Artery Stenosis in Older Adults With Different Levels of Glucose Metabolism: A Real-World Retrospective Clinical Study

**DOI:** 10.31083/RCM45886

**Published:** 2025-12-16

**Authors:** Qinyu Sun, Yifan Deng, Yuan Zhang, Zhen Fang, Jun Ji, Shenghu He, Jing Zhang

**Affiliations:** ^1^Department of Cardiology, Northern Jiangsu People’s Hospital Affiliated to Yangzhou University, 225001 Yangzhou, Jiangsu, China; ^2^Department of Cardiology, Northern Jiangsu People’s Hospital, 225001 Yangzhou, Jiangsu, China

**Keywords:** coronary heart disease, C-reactive protein–triglyceride glucose index, predictive value, predictive model, older adults

## Abstract

**Background:**

Coronary heart disease (CHD), one of the most severe cardiovascular conditions, poses a significant threat to the health and survival of older adults. Numerous studies have confirmed that diabetes, inflammation, and dyslipidemia are key risk factors for CHD. However, the relationship between the C-reactive protein–triglyceride glucose index (CTI) and CHD risk in older adults across different glucose metabolism statuses remains unexplored. Thus, this study aimed to investigate the correlation between the CTI and CHD risk in older adults with varying glycemic statuses.

**Methods:**

Patients aged ≥60 years, who underwent coronary angiography between January 2019 and December 2023, were enrolled. A diagnosis of CHD was performed when the coronary angiography demonstrated ≥50% stenosis in at least one major epicardial vessel. Demographic characteristics, medical history, laboratory data, and procedural records were systematically collected. Least absolute shrinkage and selection operator (Lasso) and multivariate logistic regression identified potential predictors. Receiver operating characteristic (ROC) curves were employed to assess the clinical value of CTI in predicting CHD risk. A restricted cubic spline (RCS) was used to examine all nonlinear relationships. A nomogram for the occurrence of CHD in older adults was constructed, and a subgroup analysis was performed.

**Results:**

A total of 1204 patients were included (919 diagnosed with CHD, 285 non-CHD (NCHD) controls). The CTI was identified as an independent risk factor for CHD (odds ratio (OR) = 4.88, 95% confidence interval (CI): 3.59–6.62). The CTI, analyzed both as a continuous and categorical variable, showed significant associations with CHD incidence across various adjusted models. The RCS analysis across different glucose metabolism statuses revealed a nonlinear relationship between the CTI and coronary artery stenosis severity in the overall population. The nomogram model based on multivariate logistic regression demonstrated good predictive accuracy for CHD in older adults.

**Conclusion:**

A positive correlation exists between the CTI and both CHD risk and the severity of coronary stenosis in older adults.

## 1. Introduction

Coronary heart disease (CHD), also referred to as ischemic heart disease, is a life-threatening cardiovascular disorder pathologically characterized by an imbalance between myocardial oxygen demand and coronary blood supply. Significant reduction in coronary perfusion or impairment of vasomotor function may lead to sustained myocardial hypoxia, thereby precipitating acute clinical events such as myocardial infarction [1]. Epidemiologic studies indicate that middle-aged and elderly populations exhibit elevated incidence and mortality rates of CHD, with prevalence exceeding 27.8% among adults aged over 60 years [2]. Notably, age-related physiological decline heightens susceptibility to metabolic abnormalities in this demographic. This process is typically accompanied by insulin resistance (IR), which exacerbates systemic metabolic dysregulation and contributes to a cascade of pathophysiological alterations—including endothelial dysfunction, chronic inflammation, and platelet hyperactivation—all of which collectively amplify cardiovascular risk [3]. Furthermore, elderly CHD patients typically demonstrate a higher burden of comorbidities and consequently experience poorer clinical outcomes compared to their younger counterparts.

IR is a pathophysiological state characterized by diminished insulin sensitivity in peripheral tissues, leading to impaired glucose uptake and utilization. Substantial evidence has established strong associations between IR and the pathogenesis of various diseases, including ischemic stroke [4], sepsis [5], and multiple cardiovascular disorders [6]. Notably, the triglyceride-glucose index (TyG), an emerging biomarker for IR assessment, has been demonstrated to correlate significantly with arterial elasticity, the extent of coronary atherosclerosis, and neurological deficit scores in patients with cerebral infarction [7]. Chronic inflammation has been identified as a critical contributor to CHD pathogenesis. Inflammation-induced endothelial dysfunction promotes subendothelial lipoprotein retention, leukocyte recruitment, and enhanced vascular permeability mediated by platelet activation [8], collectively facilitating fibroatheroma development. In this context, Ruan *et al*. [9] innovatively proposed the C-reactive protein-triglyceride-glucose index (CTI) in 2022. This composite biomarker integrates inflammatory markers (C-reactive protein) with parameters of IR (TyG index). Although initially applied for prognostic evaluation in oncology, this multidimensional approach offers novel perspectives for assessing metabolic and inflammation-related diseases.

Studies have demonstrated that IR is independently associated with cardiovascular events in both diabetic (DM) and non-diabetic (NDM) individuals. Furthermore, accumulating evidence indicates that IR is linked to subclinical vascular damage, which manifests as functional and structural alterations of the arterial wall that cannot be fully explained by conventional risk factors [10]. Currently, coronary angiography remains the gold standard for clinically diagnosing CHD and assessing the severity of coronary artery lesions. Based on angiographic findings, this study aims to quantify the extent of coronary artery lesions, identify factors influencing CHD development in elderly patients, and investigate the association between CTI and both CHD prevalence and severe coronary lesions across varying glucose metabolism statuses. The ultimate objective is to establish a practical and efficient screening tool to facilitate early identification of elderly CHD patients and high-risk individuals.

## 2. Methods and Materials

### 2.1 Study Subjects

**Fig. 1.**
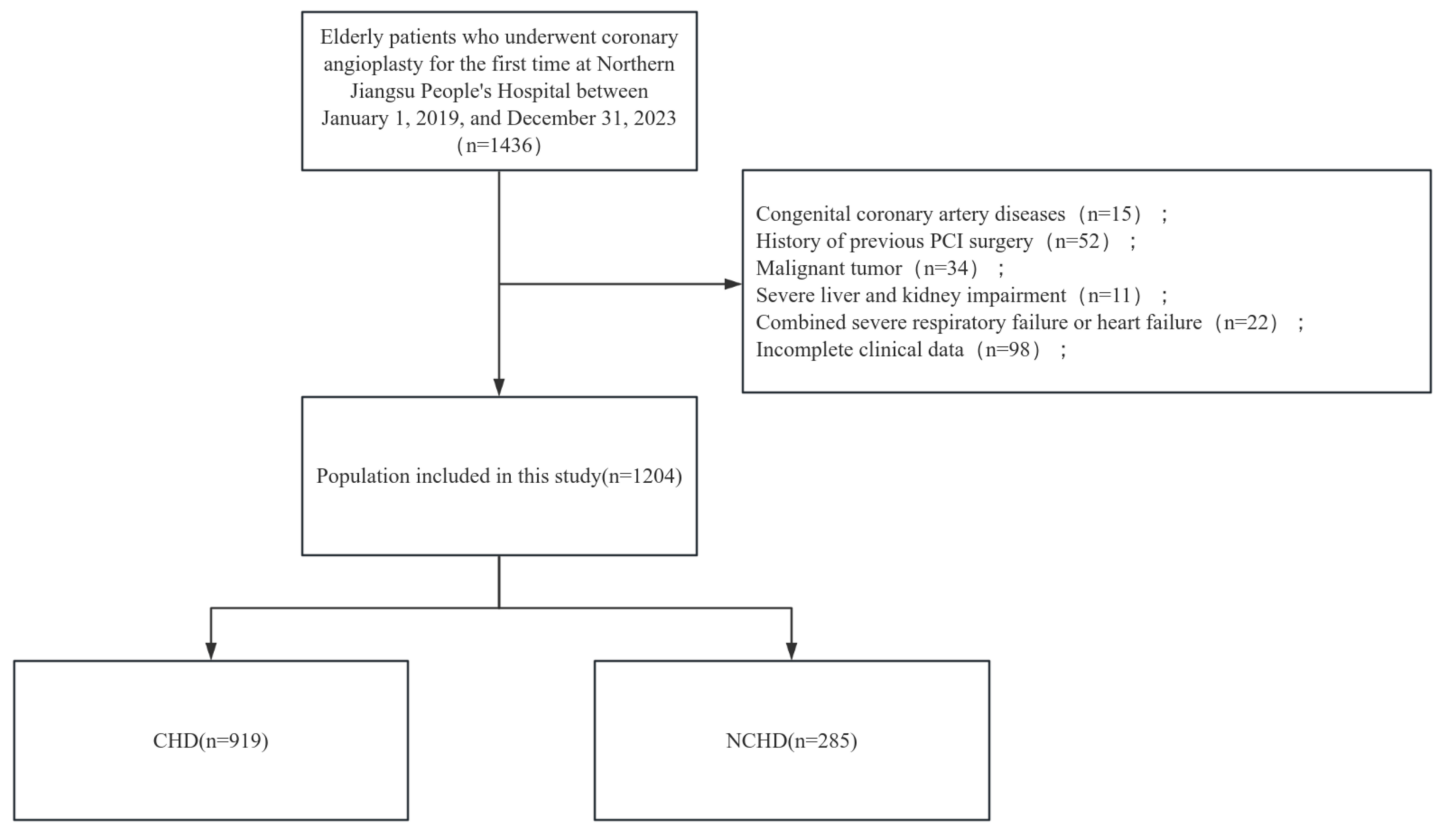
**Flowchart of grouping of patients**. CHD, coronary heart disease; NCHD, non-CHD; PCI, percutaneous coronary intervention.

This retrospective study investigated the association between CTI and CHD in elderly patients undergoing coronary angiography at Northern Jiangsu People’s Hospital from January 2019 to December 2023. Participants were rigorously selected based on predefined inclusion/exclusion criteria and thorough data completeness verification. Using quantitative coronary angiography as the diagnostic standard, patients were stratified into two groups: the CHD group with ≥50% epicardial coronary artery stenosis and the non-CHD group with <50% stenosis (Fig. 1) [11], with the 50% threshold reflecting clinically significant coronary obstruction. The study design ensured methodological rigor through standardized patient selection and objective angiographic assessment.

Inclusion criteria: (1) age ≥60 years; (2) have typical angina symptoms and choose to undergo coronary angiography elective admission; (3) complete clinical data available; (4) no hereditary or familial disease history.

Exclusion criteria: (1) severe hepatic dysfunction (defined as aspartate aminotransferase [AST] or alanine aminotransferase [ALT] levels ≥3 times the upper limit of normal) or chronic kidney disease (CKD) stages 4–5 (glomerular filtration rate [GFR] <30 mL/min); (2) previous history of cardiac surgery, severe cardiovascular or respiratory disease (New York Heart Association (NYHA) cardiac function class III.–IV., cardiogenic shock or respiratory failure, etc.); (3) other systemic serious diseases (such as hyperthyroidism, active infection, malignant tumors, autoimmune diseases, or hematological diseases); (4) patients with familial hyperlipidemia.

### 2.2 Data Collection and Definition

Baseline data on patients were collected through the electronic medical record system, including gender, age, smoking history, alcohol consumption history, history of hypertension, and history of diabetes.

Relevant criteria and definitions:

(1) Smoking history is defined as smoking more than one cigarette per day, with the habit persisting or accumulating for more than six months;

(2) Alcohol consumption history is defined as daily ethanol intake of at least 20 g for men and at least 10 g for women;

(3) Hypertension is diagnosed when blood pressure is measured three times on non-consecutive days, with systolic blood pressure ≥140 mmHg (1 mmHg = 0.133 kPa) and/or diastolic blood pressure ≥90 mmHg;

(4) Diabetes mellitus (DM): Diagnosis is confirmed if any of the following criteria are met: (1) the patient reports currently using antidiabetic medications; (2) fasting plasma glucose (FPG) ≥126 mg/dL (7.0 mmol/L); (3) 2-hour blood glucose ≥200 mg/dL (11.1 mmol/L) during an oral glucose tolerance test (OGTT). Prediabetes mellitus (Pre-DM): Fasting plasma glucose 100–125 mg/dL (5.6–6.9 mmol/L) or glycated hemoglobin (HbA1c) 5.7–6.4%.

(5) The CTI index is calculated using the following formula [12]: CTI = 0.412 × Ln (C-reactive protein (CRP) [mg/L]) + Ln (Triglycerides (TG) [mg/dL] × FPG [mg/dL])/2.

Patients were promptly arranged for venous blood sample collection upon admission to measure hemoglobin, white blood cells, and other routine blood parameters. After a 12-hour fast, venous blood was collected again the following morning to measure fasting blood glucose, lipid levels, and other biochemical parameters. The left ventricular ejection fraction was precisely assessed using the two-plane Simpson method.

### 2.3 Analysis of Coronary Artery Stenosis Severity

All CHD patients were evaluated for the degree of coronary stenosis using the Gensini score [13], and all patients’ coronary angiographic findings were evaluated and recorded by 2 or more specialized coronary interventionalists.

### 2.4 Statistical Analysis 

Statistical analyses were performed using SPSS ver. 27 (IBM, Armonk, NY, USA) and R 4.1.1 (R Foundation for Statistical Computing, Vienna, Austria). The normality of continuous variables was assessed using the Kolmogorov-Smirnov test. Normally distributed data were presented as standard deviation ($\bar{x}$ ± s) and analyzed using the *t*-test, while non-normally distributed data were expressed as median (Q_1_, Q_3_) and analyzed using the Mann-Whitney U test. Categorical variables were described as frequencies (percentages) and compared using χ^2^ or Fisher’s exact tests as appropriate.

Least absolute shrinkage and selection operator (Lasso) and multivariate logistic regression analyses were employed to identify independent risk factors for CHD in elderly patients. The predictive performance of CTI was evaluated using receiver operating characteristic (ROC) curve analysis, with area under the curve (AUC) and 95% confidence intervals (CI) reported. Multiple logistic regression models (including unadjusted and two adjusted models) were constructed to assess the association between CTI (analyzed both as continuous and quartile-categorized variables) and CHD risk, with results expressed as odds ratios (ORs) and 95% CIs.

A nomogram prediction model was developed based on the regression results and validated through (1) calibration using the Hosmer-Lemeshow test and internal validation via bootstrap resampling (1000 iterations), (2) discrimination assessment using ROC curve analysis, and (3) clinical utility evaluation via decision curve analysis (DCA). Additional ROC analyses were conducted to evaluate the predictive value of CTI for CHD across different glycemic statuses. Subgroup analyses stratified by gender, smoking status, alcohol consumption, and hypertension were performed to examine potential interaction effects on the CTI-CHD association. Coronary stenosis severity was quantified using Gensini scores, and the nonlinear relationship between CTI and stenosis degree was analyzed using restricted cubic spline (RCS) regression models.

*p* value of <0.05 is considered statistically significant.

## 3. Results

### 3.1 Comparison of Baseline Data Between CHD and NCHD Groups

The study comprised 1204 participants, with 919 (76.33%) in the CHD group and 285 (23.67%) in the NCHD group. Significant intergroup differences (*p* < 0.05) were observed in multiple parameters: gender distribution, smoking status, alcohol consumption history, prevalence of hypertension and diabetes mellitus, CTI values, body mass index (BMI), fasting blood glucose, glycosylated hemoglobin A1c, hemoglobin levels, white blood cell count, neutrophil count, lymphocyte count, monocyte count, triglyceride levels, high-density lipoprotein, low-density lipoprotein, lipoprotein (a), apolipoprotein A1, serum albumin, uric acid and C-reactive protein. In contrast, no statistically significant differences (*p* > 0.05) were found for age, systolic/diastolic blood pressure, left ventricular ejection fraction (LVEF), platelet count, total cholesterol, apolipoprotein B, or serum potassium levels (Table 1).

**Table 1.** **Comparison of baseline data between CHD and NCHD groups**.

Variables		Total (n = 1204)	CHD (n = 919)	NCHD (n = 285)	Statistic	*p*
Age [M (Q_1_, Q_3_), years]		75 (67, 83)	74 (68, 82)	76 (67, 83)	Z = –0.33	0.738
BMI [M (Q_1_, Q_3_), kg/m^2^]		25.35 (22.71, 27.67)	24.65 (22.43, 26.72)	25.56 (22.86, 27.80)	Z = –3.46	<0.001
Systolic blood pressure [mmHg, M (Q_1_, Q_3_)]		132 (122, 147)	132 (120, 147)	133 (124, 147)	Z = –1.40	0.160
Diastolic blood pressure [mmHg, M (Q_1_, Q_3_)]		79 (70, 87)	78 (70, 87)	79 (71, 85)	Z = –0.15	0.877
BMI [kg/m^2^, M (Q_1_, Q_3_)]		25.35 (22.71, 27.67)	25.56 (22.86, 27.80)	24.65 (22.43, 26.72)	Z = –3.46	<0.001
LVEF [M (Q_1_, Q_3_), (%)]		56 (50, 61)	56 (50, 61)	56 (50, 63)	Z = –1.84	0.065
Fasting blood glucose [M (Q_1_, Q_3_), mmol/L]		6.06 (5.10, 7.80)	6.27 (5.17, 8.00)	5.64 (5.00, 7.16)	Z = –3.42	<0.001
Glycosylated hemoglobin, Type A1C [M (Q_1_, Q_3_), %]		6.72 (5.80, 6.90)	6.72 (6.00, 6.80)	6.00 (5.50, 7.20)	Z = –5.87	<0.001
Hemoglobin [M (Q_1_, Q_3_), g/L]		137.08 (126.00, 149.00)	137.00 (124.00, 149.00)	139.00 (130.00, 150.00)	Z = –3.02	0.003
White blood cells counts [M (Q_1_, Q_3_), ×10^9^/L]		7.39 (5.87, 9.57)	8.08 (6.28, 10.46)	5.90 (4.96, 6.89)	Z = –13.87	<0.001
Neutrophil count [M (Q_1_, Q_3_), ×10^9^/L]		5.11 (3.66, 7.22)	5.82 (4.11, 7.97)	3.66 (2.96, 4.64)	Z = –14.19	<0.001
Lymphocyte count [M (Q_1_, Q_3_), ×10^9^/L]		1.55 (1.20, 2.00)	1.53 (1.14, 1.99)	1.60 (1.33, 2.04)	Z = –2.89	0.004
Monocyte count [M (Q_1_, Q_3_), ×10^9^/L]		0.46 (0.33, 0.62)	0.51 (0.37, 0.66)	0.34 (0.27, 0.43)	Z = –12.65	<0.001
Platelet count [M (Q_1_, Q_3_), ×10^9^/L]		189.00 (153.00, 228.00)	189.00 (152.00, 227.00)	189.00 (158.00, 232.00)	Z = –0.62	0.536
Triglycerides [M (Q_1_, Q_3_), mmol/L]		1.56 (1.11, 2.29)	1.59 (1.15, 2.32)	1.44 (1.00, 2.17)	Z = –2.00	0.045
Total cholesterol [M (Q_1_, Q_3_), mmol/L]		4.36 (3.66, 4.91)	4.34 (3.67, 4.89)	4.38 (3.57, 5.00)	Z = –0.20	0.841
High-density lipoprotein [M (Q_1_, Q_3_), mmol/L]		1.06 (0.89, 1.24)	1.03 (0.87, 1.23)	1.15 (0.96, 1.43)	Z = –6.73	<0.001
Low-density lipoprotein [M (Q_1_, Q_3_), mmol/L]		2.85 (2.24, 3.68)	2.92 (2.34, 3.94)	2.64 (1.89, 3.16)	Z = –6.52	<0.001
Lipoprotein (a) [M (Q_1_, Q_3_), mg/L]		178.00 (111.33, 276.70)	188.40 (119.90, 285.65)	143.70 (81.40, 250.80)	Z = –4.80	<0.001
Apolipoprotein A1 [M (Q_1_, Q_3_), g/L]		1.27 (1.11, 1.43)	1.22 (1.08, 1.37)	1.41 (1.27, 1.59)	Z = –11.56	<0.001
Apolipoprotein B [M (Q_1_, Q_3_), g/L]		0.90 (0.75, 1.05)	0.91 (0.75, 1.05)	0.89 (0.74, 1.03)	Z = –1.03	0.301
C-reactive protein [M (Q_1_, Q_3_), mg/L]		4.35 (0.96, 12.59)	10.17 (2.11, 12.59)	0.76 (0.38, 1.47)	Z = –18.58	<0.001
Albumin [M (Q_1_, Q_3_), g/L]		42.21 (39.00, 45.50)	41.10 (38.10, 44.30)	45.20 (43.00, 47.60)	Z = –13.22	<0.001
Uric acid [M (Q_1_, Q_3_), µmol/L]		326.60 (265.88, 390.22)	332.78 (270.40, 394.30)	303.20 (252.10, 362.90)	Z = –3.89	<0.001
Potassium [M (Q_1_, Q_3_), mmol/L]		3.87 (3.67, 4.13)	3.87 (3.67, 4.13)	3.87 (3.68, 4.12)	Z = –0.88	0.378
CTI [M (Q_1_, Q_3_)]		5.49 (4.83, 5.97)	5.69 (5.19, 6.07)	4.68 (4.28, 5.16)	Z = –17.03	<0.001
Gender [n, (%)]					χ^2^ = 86.31	<0.001
	Female	392 (32.56)	235 (25.57)	157 (55.09)		
	Male	812 (67.44)	684 (74.43)	128 (44.91)		
Smoking [n, (%)]					χ^2^ = 39.17	<0.001
	No	629 (52.24)	434 (47.23)	195 (68.42)		
	Yes	575 (47.76)	485 (52.77)	90 (31.58)		
Drinking [n, (%)]					χ^2^ = 8.74	0.003
	No	928 (77.08)	690 (75.08)	238 (83.51)		
	Yes	276 (22.92)	229 (24.92)	47 (16.49)		
Hypertension [n, (%)]					χ^2^ = 4.03	0.045
	No	426 (35.38)	311 (33.84)	115 (40.35)		
	Yes	778 (64.62)	608 (66.16)	170 (59.65)		
Diabetes, n (%)					χ^2^ = 64.86	<0.001
	DM	790 (65.61)	642 (69.86)	148 (51.93)		
	NDM	199 (16.53)	108 (11.75)	91 (31.93)		
	Pre-DM	215 (17.86)	169 (18.39)	46 (16.14)		

BMI, body mass index; LVEF, left ventricular ejection fraction; CTI, C-reactive protein-triglyceride-glucose index; DM, diabetes mellitus; NDM, non-diabetes mellitus; Pre-DM, prediabetes mellitus.

### 3.2 Logistic Regression Analysis of CHD Risk

**Fig. 2.**
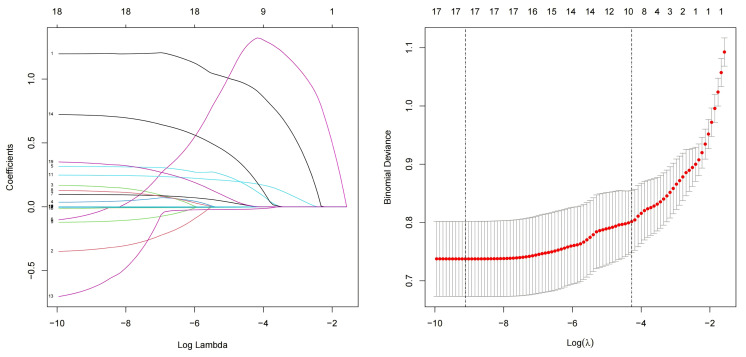
**Lasso regression**. Lasso, least absolute shrinkage and selection operator.

To address multicollinearity, with CHD incidence as the dependent variable, the statistically significant indicators in Table 1—gender (male), smoking history, drinking history, hypertension and diabetes status, CTI, BMI, fasting blood glucose, glycated hemoglobin A1c, hemoglobin, white blood cell count, neutrophil count, lymphocyte count, monocyte count, triglycerides, high-density lipoprotein, low-density lipoprotein, lipoprotein (a), apolipoprotein A1, albumin, and uric acid as independent variables in a Lasso regression analysis. The results showed that 17 factors were associated with the incidence of CHD in the elderly: male gender, smoking history, alcohol abuse history, hypertension history, BMI, fasting blood glucose, glycated hemoglobin A1c, white blood cell count, monocyte count, low-density lipoprotein (LDL), lipoprotein (a), uric acid, CTI, non-diabetes, high-density lipoprotein (HDL), apolipoprotein A1, and albumin, as shown in Fig. 2.

Meaningful indicators identified from Lasso regression were incorporated into the multivariate logistic regression. The results showed that male gender, CTI, BMI, and white blood cell count were independent risk factors for CHD, while non-diabetic mellitus (NDM) was an independent protective factor (Table 2) (*p* < 0.05).

**Table 2.** **Multivariable logistic regression**.

Variables	β	S.E	Z	*p*	OR (95% CI)
Male	1.31	0.19	6.80	<0.001	3.71 (2.54~5.42)
NDM	–0.73	0.24	–3.02	0.003	0.48 (0.30~0.78)
CTI	1.58	0.16	10.16	<0.001	4.88 (3.59~6.62)
White blood cells	0.25	0.05	5.45	<0.001	1.29 (1.18~1.41)
Low-density lipoprotein	0.67	0.09	7.54	<0.001	1.96 (1.64~2.33)

S.E., standard error; Z, Z-score; OR, odds ratio; CI, confidence interval.

### 3.3 Correlation Between CTI and the Occurrence of CHD

When CTI is treated as a continuous variable, it shows significant correlation in all three models (*p* < 0.05). When CTI is treated as a categorical variable, the Q_1_ group consists of 303 individuals (CTI ≤4.83), the Q_2_ group consists of 312 individuals (4.83 < CTI ≤ 5.49), Q_3_) group: 294 individuals (5.49 < CTI ≤ 5.97), and Q_4_) group: 295 individuals (CTI >5.97), the risk of CHD increased gradually with the rise in CTI quartiles (*p* < 0.05) (Table 3).

**Table 3.** **Association between CTI and CHD (logistic regression)**.

Variables		Model 1		Model 2		Model 3	
		OR (95% CI)	*p*	OR (95% CI)	*p*	OR (95% CI)	*p*
CTI		6.273 (4.916~8.003)	<0.001	5.842 (4.504~7.578)	<0.001	4.272 (3.237~5.637)	<0.001
	Q_1_	-		-		-	
	Q_2_	2.695 (1.937~3.750)	<0.001	2.623 (1.839~3.741)	<0.001	1.792 (1.215~2.643)	0.003
	Q_3_	15.338 (9.321~25.239)	<0.001	13.202 (7.860~22.176)	<0.001	7.506 (4.337~12.991)	<0.001
	Q_4_	68.432 (27.482~170.399)	<0.001	62.268 (24.574~157.784)	<0.001	33.898 (13.000~88.391)	<0.001

OR, odds ratio; CI, confidence interval. Model 1: Crude. Model 2: Adjust: Gender, Diabetes. Model 3: Adjust: Gender, Diabetes, BMI, White blood cells, Lymphocyte count, Low-density lipoprotein.

### 3.4 Predictive Value of CTI for CHD Risk at Different Levels of Glucose Metabolism

**Fig. 3.**
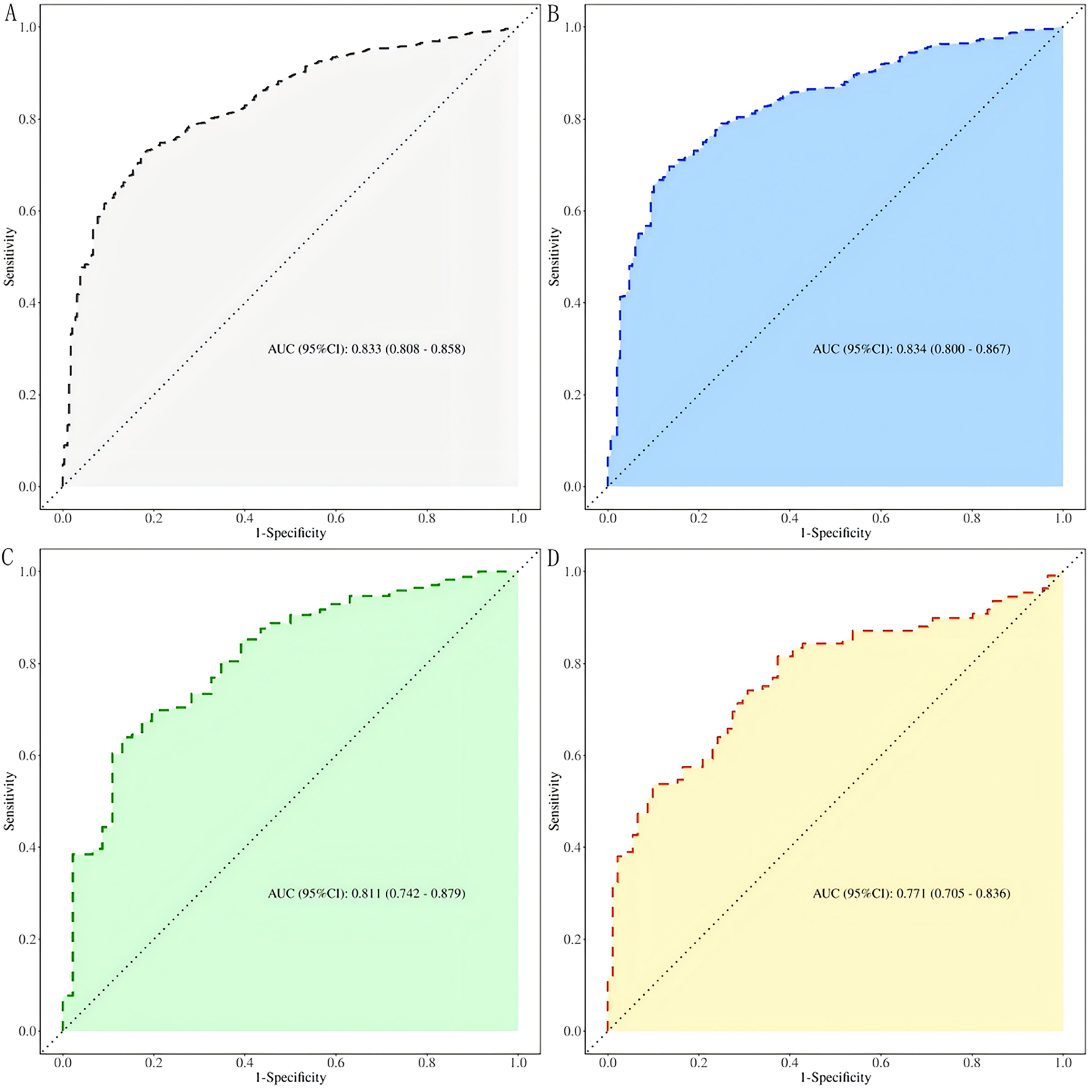
**ROC curves of the predictive value of CTI for CHD in the elderly at different levels of glucose metabolism**. (A) All patients; (B) DM; (C) Pre-DM; (D) NDM. ROC, receiver operating characteristic.

ROC curve analysis demonstrated that CTI consistently exhibited good predictive value for CHD risk across all glucose tolerance subgroups. Notably, CTI showed the highest predictive performance in the DM group, with an AUC of 0.834 (95% CI: 0.800–0.867). Among the three subgroups, CTI achieved the highest sensitivity for CHD prediction in the pre-DM population, while its specificity was highest in the NDM group (*p* < 0.05) (Table 4, Fig. 3).

**Table 4.** **Predictive value of CTI for CHD risk at different levels of glucose metabolism**.

State	AUC (95% CI)	Accuracy (95% CI)	Sensitivity (95% CI)	Specificity (95% CI)	Cut off
All patients	0.833 (0.808–0.858)	0.750 (0.725–0.774)	0.821 (0.777–0.866)	0.728 (0.699–0.757)	5.262
DM	0.834 (0.800–0.867)	0.728 (0.695–0.759)	0.865 (0.810–0.920)	0.696 (0.661–0.732)	5.480
Pre-DM	0.811 (0.742–0.879)	0.688 (0.622–0.750)	0.870 (0.772–0.967)	0.639 (0.567–0.711)	5.265
NDM	0.771 (0.705–0.836)	0.729 (0.661–0.789)	0.626 (0.527–0.726)	0.815 (0.742–0.888)	4.581

AUC, area under the curve.

### 3.5 Predictive Modeling and Evaluation

**Fig. 4.**
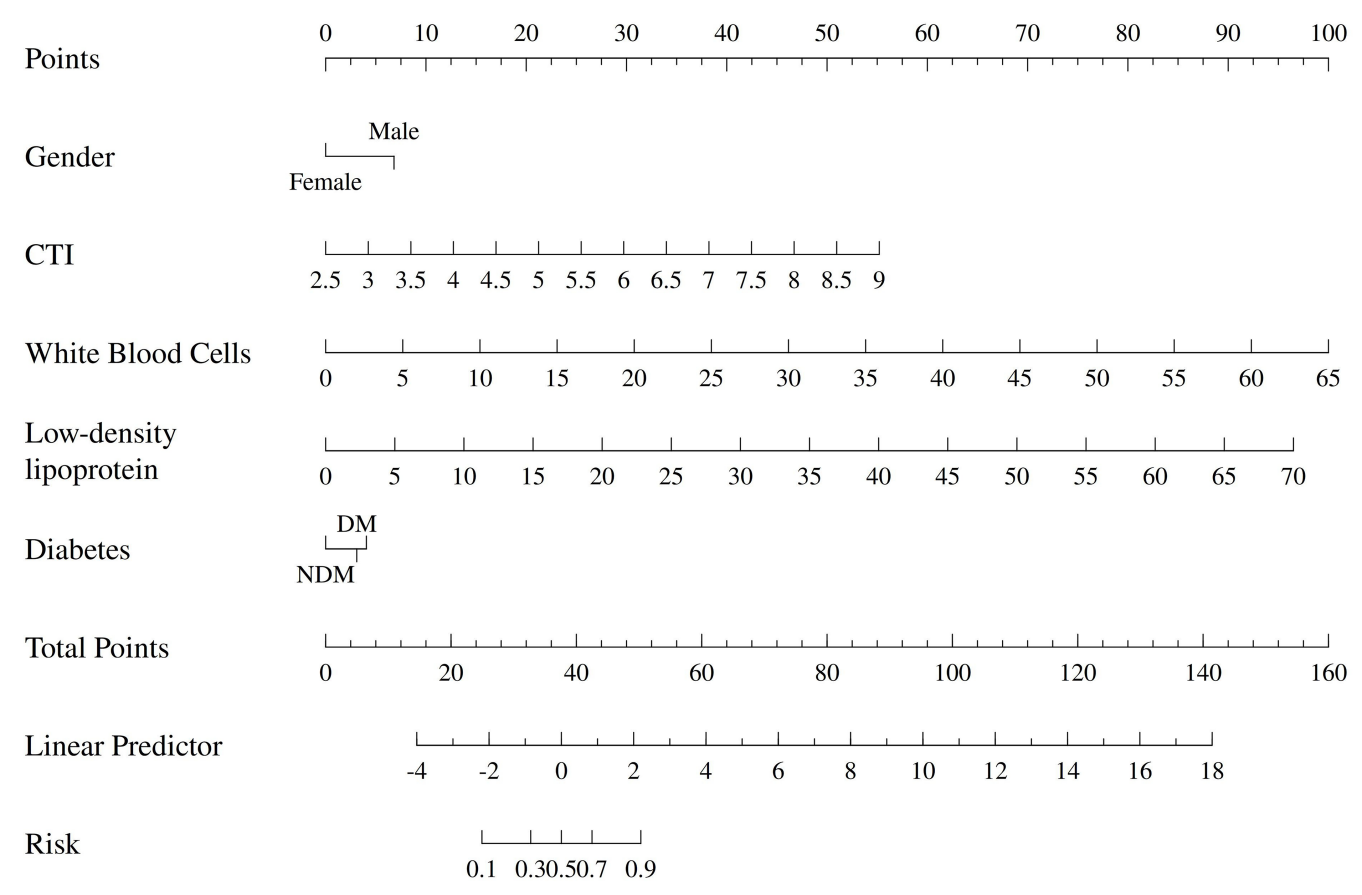
**Nomogram of the prediction model**.

Based on the results of the multivariate logistic regression analysis, a nomogram model was constructed to predict the risk of CHD in the elderly population, incorporating gender, CTI, white blood cell count, low-density lipoprotein cholesterol, and diabetic status (Fig. 4).

**Fig. 5.**
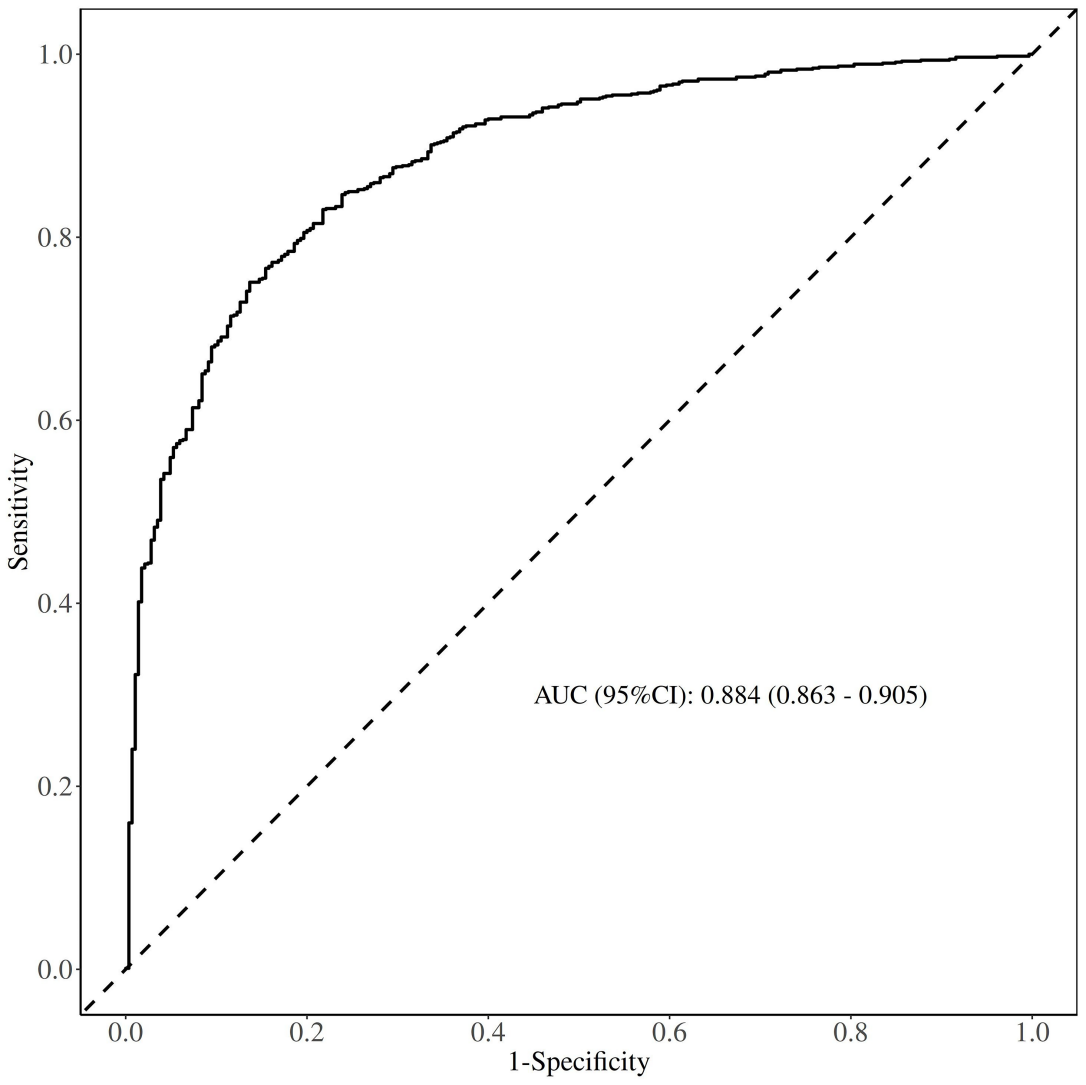
**ROC curve of the prediction model**.

**Fig. 6.**
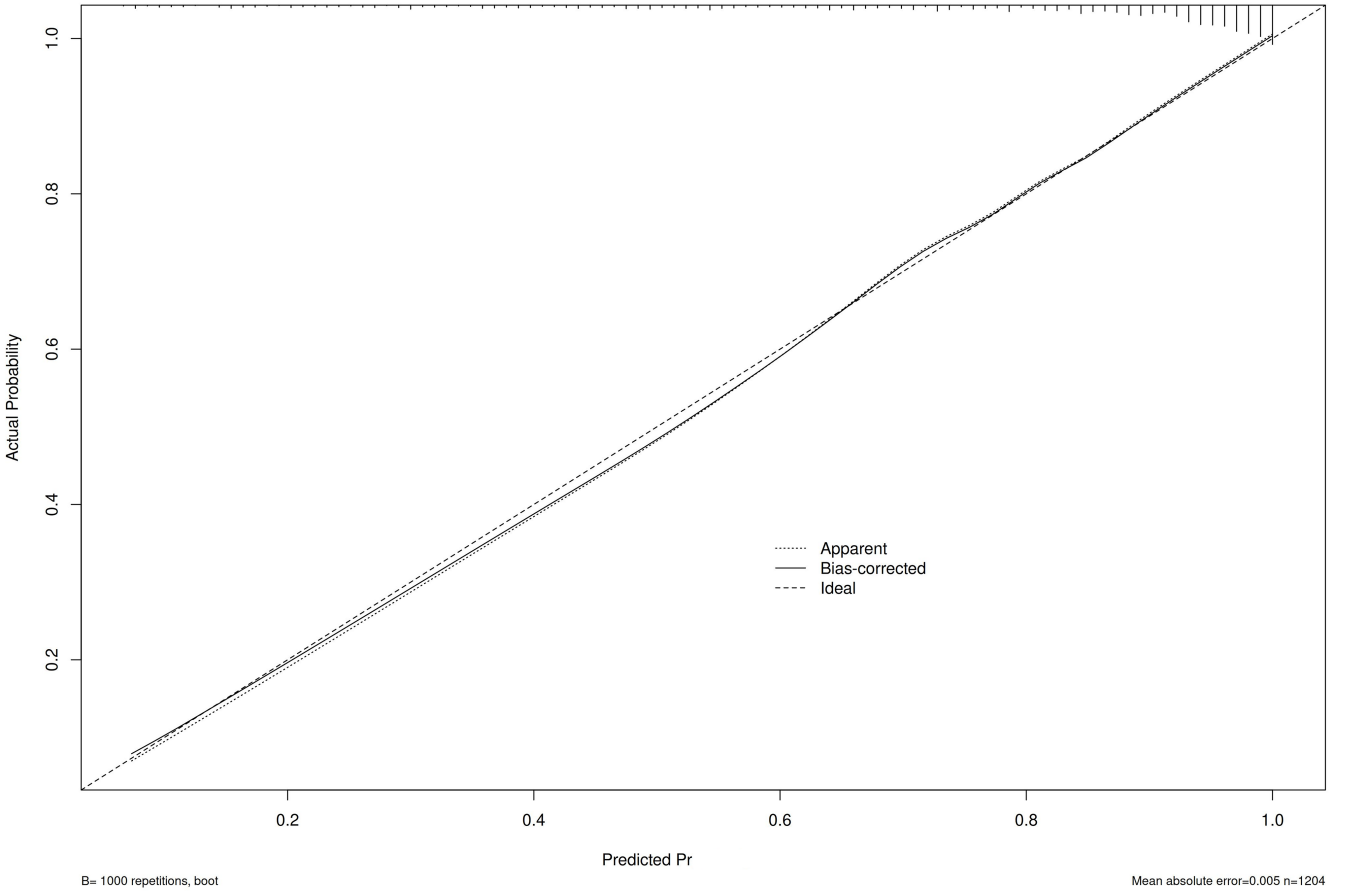
**Hosmer-Lemeshow curve of the prediction model**.

**Fig. 7.**
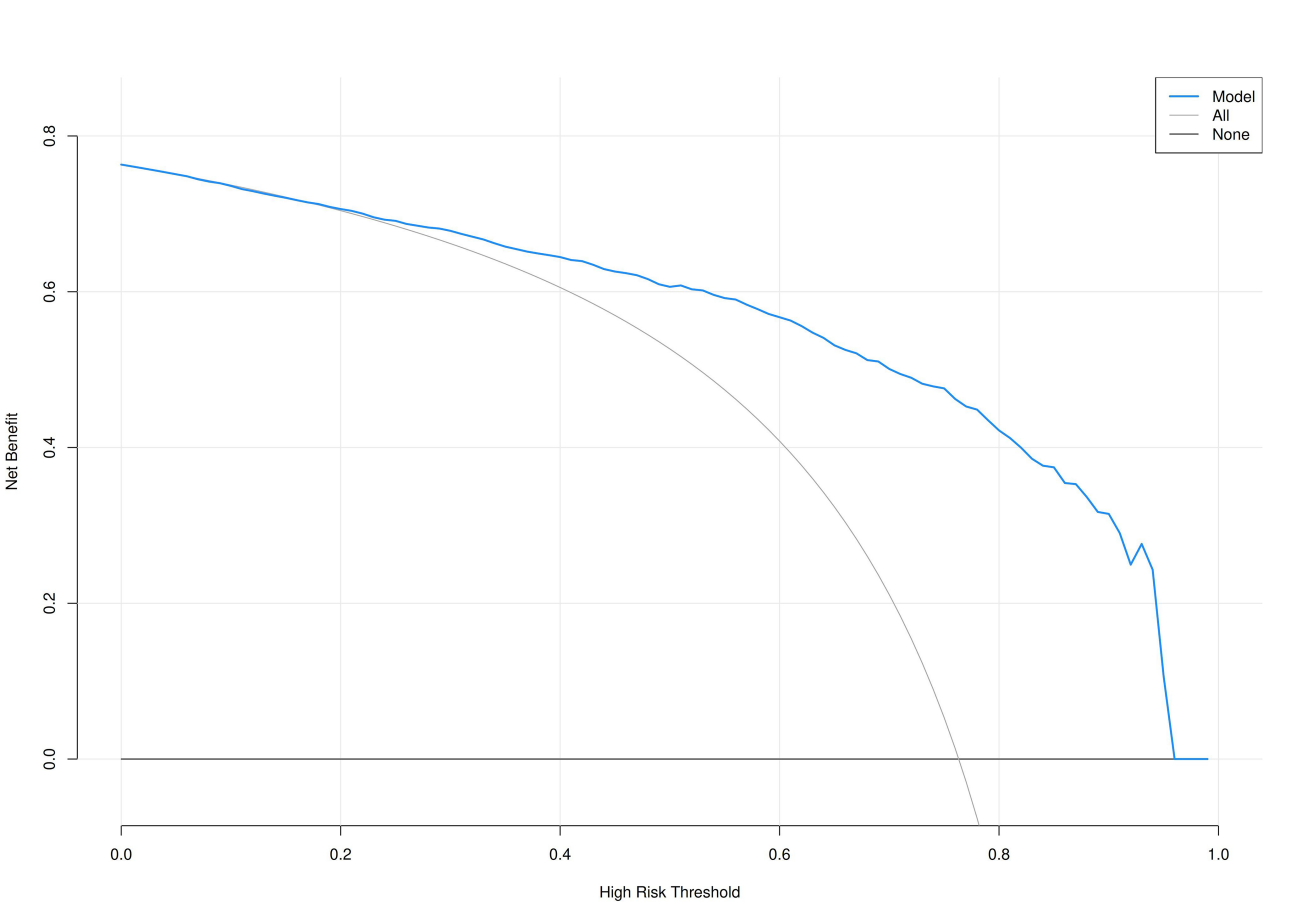
**Decision curve analysis (DCA) curve of the prediction model**.

The area under the ROC curve (AUC) of the nomogram model was 0.884 (95% CI: 0.863–0.905), indicating strong discriminative ability (Fig. 5). Internal validation was performed using the bootstrap method with 1000 resamples (B = 1000). The calibration curve showed a mean absolute error of *p* = 0.412 (>0.05), suggesting good model accuracy (Fig. 6). Additionally, DCA demonstrated favorable net benefit for patients, confirming the model’s clinical utility (Fig. 7).

### 3.6 Subgroup Analysis

**Fig. 8.**
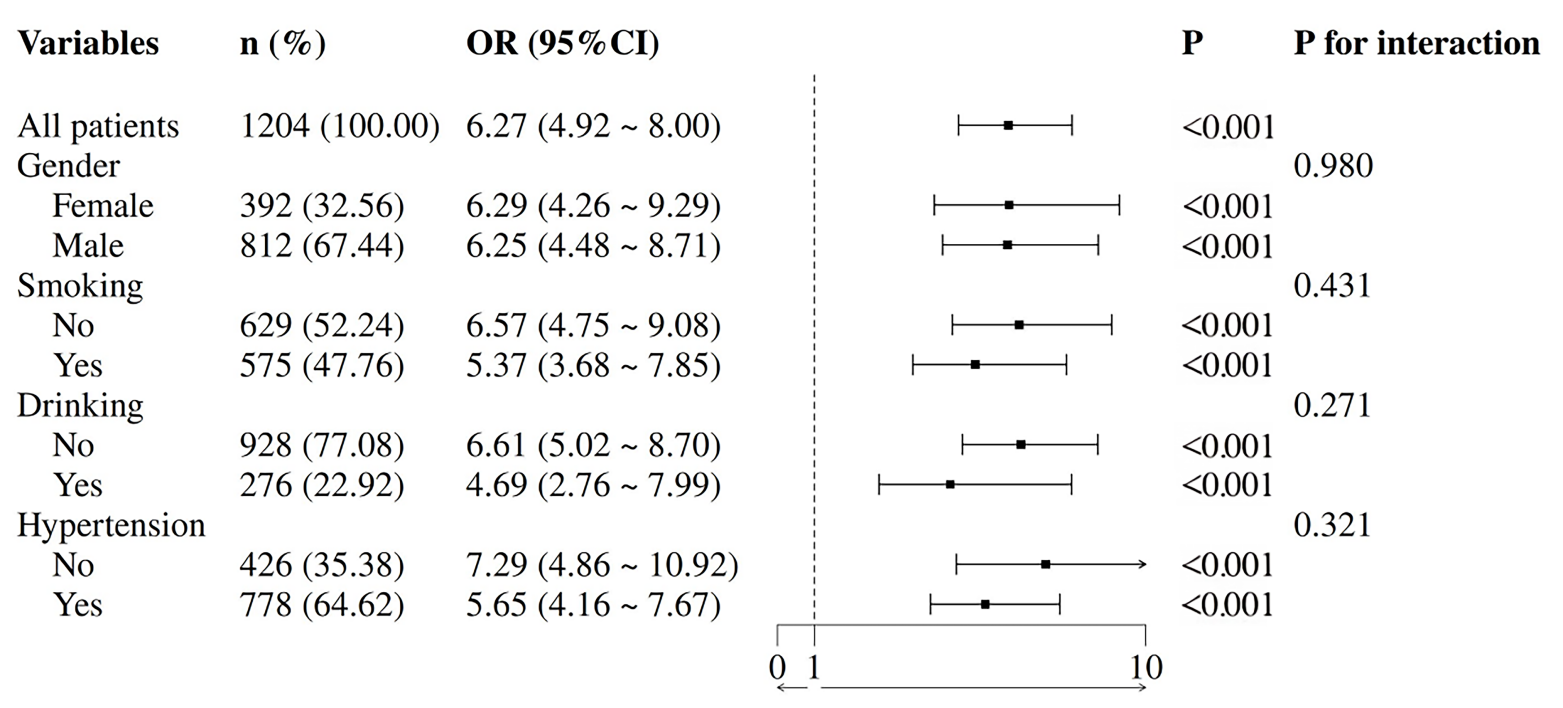
**Subgroup analysis**.

To further investigate the association between CTI and CHD risk, subgroup analyses were performed. The results demonstrated the consistent predictive efficacy of CTI for CHD risk across various stratified factors, including gender, smoking status, drinking and hypertension. A significant positive correlation between CTI and CHD risk was observed in all subgroups (*p* for interaction >0.05) (Fig. 8).

### 3.7 Correlation Between CTI and Degree of Coronary Artery Stenosis at Different Levels of Glucose Metabolism

**Fig. 9.**
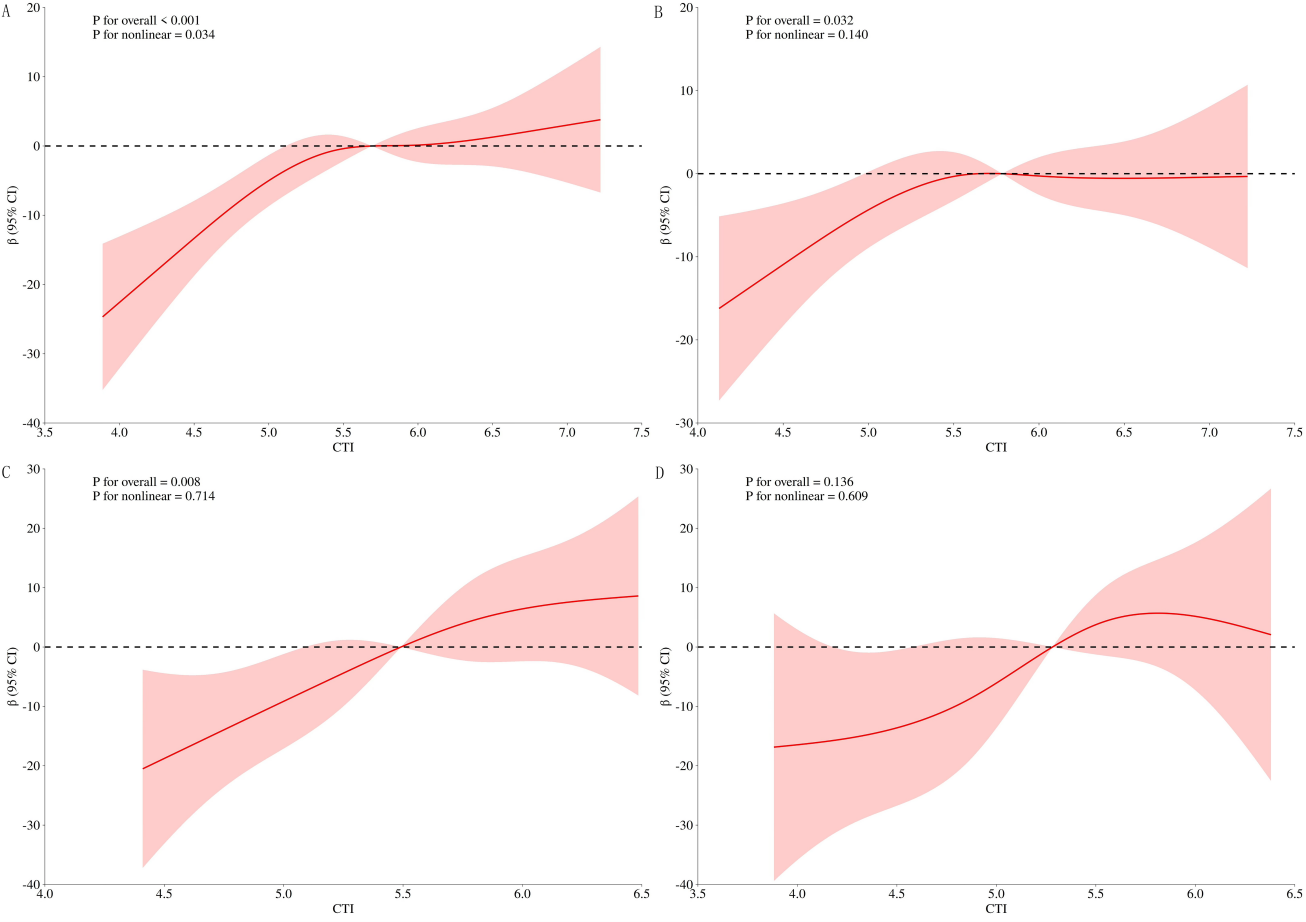
**RCS curves of CTI and coronary stenosis at different levels of glucose metabolism**. (A) All patients; (B) DM; (C) Pre-DM; (D) NDM. RCS, restricted cubic spline.

In Model 3, restricted cubic spline regression analysis across different glucose metabolism statuses revealed a nonlinear relationship between CTI and coronary artery stenosis severity in the overall population. However, in the NDM subgroup, no significant association was observed between CTI and stenosis degree (Fig. 9).

## 4. Discussion

Our findings demonstrate that CTI is an independent risk factor for CHD in elderly populations (OR = 4.88, 95% CI: 3.59–6.62). As a novel composite biomarker integrating CRP and TyG index, CTI provides a holistic assessment of both inflammatory status and IR severity. Unlike CRP or TyG alone, which reflect isolated pathophysiological dimensions, CTI captures synergistic or compensatory interactions between inflammation and IR, thereby improving risk stratification accuracy. The complementary roles of these biomarkers—reflecting inflammatory activity and metabolic dysfunction, respectively—enhance the identification of high-risk profiles while mitigating the limitations of individual biomarker variability. This mechanistic rationale is corroborated by the significant association between CTI and CHD observed in our study, which aligns with previous reports by Xu *et al*. [14] indicating a 1.357-fold increase in CHD risk per standard deviation rise in CTI. Importantly, CTI represents a readily accessible, cost-effective clinical tool that holistically reflects inflammatory-metabolic dysregulation. Its simplicity and robustness support its potential utility in routine screening for elevated CHD risk in elderly populations.

Our study further revealed that CTI remained significantly associated with CHD incidence across different glucose metabolic states. When analyzed as a continuous variable, CTI demonstrated a clear dose-response relationship with CHD occurrence in elderly populations—a trend that persisted when CTI was categorized into quartiles (Q1–Q4). ROC curve analysis consistently confirmed the discriminative capacity of CTI for CHD risk stratification, irrespective of glycemic status. Notably, CTI exhibited superior predictive performance in patients of DM (AUC: 0.834), suggesting an enhanced prognostic utility under conditions of pronounced metabolic dysregulation. Both subgroup analyses and RCS curves consistently indicated a strong positive association between CTI and CHD risk in elderly individuals. This relationship remained significant after comprehensive adjustment for multiple confounders and proved stable across all predefined stratification variables. Furthermore, CTI showed significant positive correlations with the severity of coronary artery stenosis, particularly among patients with DM and those with pre-DM.

Our findings indicate that elderly individuals exhibit significantly reduced vascular elasticity and more impaired endothelial function compared to younger populations. These age-related structural and functional changes, compounded by chronic low-grade inflammation and elevated oxidative stress, collectively promote lipid deposition and accelerate atherosclerotic plaque formation. In patients with DM or pre-DM, disordered glucose metabolism further exacerbates atherosclerosis through multiple synergistic pathways. A self-amplifying cycle emerges involving hyperglycemia-induced endothelial dysfunction, dyslipidemia, and IR-related metabolic dysregulation. Importantly, aging potentiates IR through heightened inflammatory responses and oxidative stress, while IR in turn aggravates vascular dysfunction via endothelial impairment and enhanced inflammation—establishing a pathogenic “aging–IR–atherosclerosis” triad that drives accelerated CHD progression [2]. At the mechanistic level, insulin-resistant adipose tissue dysfunction promotes systemic inflammation via increased release of proinflammatory cytokines (e.g., TNF-α, IL-6), fostering a microenvironment conducive to monocyte infiltration and foam cell formation, which are critical to atherosclerotic lesion development [15].

These mechanisms account for the superior predictive performance of CTI in DM patients, as diabetic metabolic dysregulation likely amplifies the synergistic vascular damage associated with both aging and CTI. The lack of a significant CTI–CHD correlation in normoglycemic individuals may be attributed to: (1) the dependency of CTI’s atherogenic effects on diabetes-specific metabolic disturbances such as chronic inflammation and IR [16]; (2) preserved insulin-mediated vasoprotective mechanisms under normoglycemic conditions; and (3) compensatory hyperinsulinemia that maintains metabolic equilibrium in subjects with intact β-cell function [17]. These findings collectively suggest that the cardiovascular implications of CTI follow a threshold effect, becoming clinically discernible only when glucoregulatory impairment reaches a critical severity.

We developed a predictive nomogram model that integrates CTI with other key clinical variables. Multivariate logistic regression identified CTI, male sex, LDL cholesterol, diabetes history, and white blood cell count as independent risk factors, collectively constituting a comprehensive risk assessment system for CHD in elderly patients. This geriatric-specific model innovatively combines conventional cardiovascular risk factors [18]—such as male sex and elevated LDL—with inflammatory markers (e.g., White Blood Cells count) and the novel CTI index, which concurrently captures insulin resistance and inflammatory activity. The synergistic interplay among these multidimensional components substantially enhances the model’s discriminative capacity and predictive accuracy.

The well-established association between inflammatory biomarkers and CHD risk [19] is mechanistically substantiated in our model. Specifically, an elevated WBC count—serving as a direct marker of systemic inflammation—reflects a chronic subclinical inflammatory state that drives vascular endothelial dysfunction and promotes atherosclerotic plaque destabilization. Moreover, our model demonstrates that metabolic disturbances stemming from islet dysfunction can significantly impair cardiovascular health even during the pre-diabetes stage. This finding is consistent with clinical reports of elevated cardiovascular risk in pre-diabetic populations [20], highlighting the importance of early metabolic assessment and intervention beyond overt diabetes.

## 5. Limitations

This study has some unavoidable limitations. First, as a single-center investigation, the absence of data from other regions or countries may introduce potential selection bias. Moreover, the model was developed and validated solely within this single-center cohort, which currently restricts its broader applicability. Consequently, the generalizability of our findings to wider populations or diverse clinical settings remains to be established. In future research, we intend to conduct multi-center external validation studies to rigorously evaluate the model’s stability and performance. Additionally, the relatively short time interval between coronary angiography and CTI measurement, while ensuring data timeliness, may not adequately capture the natural progression of coronary atherosclerosis. Future studies could extend the observation period or employ longitudinal designs to further validate and contextualize our results.

## 6. Conclusion

CTI demonstrates a positive correlation with CHD risk among elderly populations across varying glucose metabolism statuses, showing significant associations with coronary artery stenosis severity in both DM and pre-DM groups. These findings suggest that CTI serves as a reliable biomarker for predicting CHD incidence and coronary stenosis severity in elderly individuals, irrespective of their glucose metabolic status.

## Availability of Data and Materials

The datasets used and analysed during the current study are available from the corresponding author on reasonable request.
